# Mobile NBM - android medical mobile application designed to help in learning how to identify the different regions of interest in the brain’s white matter

**DOI:** 10.1186/1472-6920-14-148

**Published:** 2014-07-18

**Authors:** Iskander Sánchez-Rola, Begoña García Zapirain

**Affiliations:** 1 DeustoTech Institute of Technology, University of Deusto, Bilbao, Spain

**Keywords:** Android, Mobile application, Medical education, Technology-enhanced learning, Brain, White matter, Tractography, Region of interest

## Abstract

**Background:**

One of the most critical tasks when conducting neurological studies is identifying the different regions of interest in the brain’s white matter. Currently few programs or applications are available that serve as an interactive guide in this process. This is why a mobile application has been designed and developed in order to teach users how to identify the referred regions of the brain. It also enables users to share the results obtained and take an examination on the knowledge thus learnt. In order to provide direct user-user or user-developer contact, the project includes a website and a Twitter account.

**Results:**

An application has been designed with a basic, minimalist look, which anyone can access easily in order to learn to identify a specific region in the brain’s white matter. A survey has also been conducted on people who have used it, which has shown that the application is attractive both in the student (final mean satisfaction of 4.2/5) and in the professional (final mean satisfaction of 4.3/5) environment. The response obtained in the online part of the project reflects the high practical value and quality of the application, as shown by the fact that the website has seen a large number of visitors (over 1000 visitors) and the Twitter account has a high number of followers (over 280 followers).

**Conclusions:**

Mobile NBM is the first mobile application to be used as a guide in the process of identifying a region of interest in the brain’s white matter. Although initially not many areas are available in the application, new ones can be added as required by users in their respective studies. Apart from the application itself, the online resources provided (website and Twitter account) significantly enhance users’ experience.

## Background

Much has been said recently about the use of mobile technologies in learning. PDAs (Personal Digital Assistant) [1] were the first to be used, but it has been shown that, despite them being an important addition to the field, students and specialists with no major technological barriers prefer smartphone platforms [2], as they are more fluid.

In no case can it be used to entirely replace traditional learning (in the case of students), or the exchange of knowledge amongst industry professionals, but rather it is intended to be used on a complementary basis, in order to bring greater mobility and simplicity to this area [3].

The use of mobile technology which provides immediate access to electronic resources in the health environment has been the object of study on several occasions [4-6], and the results obtained from these studies have been very positive. They have shown that the use of mobile devices could have beneficial effects on service provision processes and on the care offered [4]. If the use guidelines are followed and combined with traditional methods of study and learning, the experience is enhanced, both for the doctor involved and for the patient, as the work can be carried out more quickly and efficiently [5], avoiding any problems that may arise related to lack of knowledge.

Few applications currently exist for mobile devices related to brain analyses, and none of them explains how to define regions of interest (ROI). One of the related applications is iSurf BrainView. It is a brain tutor based on the MRI (Magnetic Resonance Imaging) automatic segmentation produced by FreeSurfer [7]. This application uses automatic segmentation to produce an automatic atlas of neuroimaging based on T1 MRI Images. It makes high quality axial, coronal, and sagittal MR images of the brain. The slider bar makes it possible to move smoothly from slice to slice. Although this application and others with similar characteristics can be of interest in order to learn the position of the different regions of the brain by using an atlas, they do not serve as a guide as to how to define a specific region of interest. In this paper, an Android mobile application for learning how to define different regions of interest in the brain’s white matter is presented. It has been developed and designed to support the conduct of brain analyses (this will be useful to anyone working in the field of tractography and white matter). It has been designed in the simplest and most intuitive way, so that anyone can have total control of the application without any major problems. Although it is considered that most medical professionals will have no problem using it, as suggested by Coulby [8], it is best not to make any assumptions about digital proficiency in the use of technology.

Two different types of examinations may be carried out by means of the application in order to verify and reinforce the knowledge learnt from the explanations previously provided (including a function to ‘tweet’ the scores obtained). Various games/examinations exist [9-11] that use this technique as a learning method, and the results obtained have been very positive. It has been proven that the knowledge obtained if a similar alternative is available, is better learnt than if such an alternative is not available [12]. As a final point, it must be noted that images of the results obtained from the analyses can be posted on the official website [13] to be shared with other professionals, which is highly beneficial [14,15].

## Methods

The application was developed using Android [16,17], as this is currently the mobile platform with the highest number of users globally, and it offers a large number of technological features. One of the things taken into account when developing the application was its complete integration with the ecosystem that users have created in their own terminal. In this way, the user can make use of their applications (Gmail, Twitter, Chrome…) and their personal accounts, quickly and simply.

The most innovative and significant technologies in current mobile technology have been in developing the app, so that the user can have an enhanced final experience. These technologies can be classified into three separate groups, which will be described below.

The first group includes verbal communication, and it can be separated internally into two subgroups. One of the technologies used is TTS (Text To Speech). By using this technology it is possible to clearly hear text, which is a great help in this case, given that users can analyse a specific brain without having to continually look away to read the procedure. Merely shaking the mobile device is enough to trigger this function and allows the user to listen to the instructions. Another of the technologies used was SR (Speech Recognition), thanks to which the user can dictate specific information requested by the application on some occasions, without the need to use the keyboard at any time.

The second group is gesture control, something that has been talked about frequently and is said to be the future of Android and mobile handsets, as it allows the easy use of an application and looks good (which is why the application was designed with this technology). Simply by drawing a pre-determined pattern, access to the different functions available is provided.

Thirdly, GPS (Global Positioning System) has also been used. This technology is currently highly relevant, given the support it provides in indicating people’s exact location. It allows the easy sharing of the location from which the data is sent (if the user so wishes), in order to publish their position on the web later.

Since not all of the Android terminals have these technologies available, two versions of the application have been created. There is a version that includes all of the functions previously outlined (compatible with the medium/high-end terminals). In addition, there is a cut-down version (for older-generation or low-end terminals), in which all of the functionalities explained are not available, but which includes all the information with respect to analysis. The application interface is optimised for a screen size of 4.65 inches. The closer the terminal screen is to this size, the better the interface will adapt.

The application also covers the creation and administration of an official Twitter account [18], an official webpage (developed and designed by using Wordpress [19]), on which images sent by users can be posted. It shows information relevant to the application (updates, compatible terminals, etc.), and news related to the world of neuro-imaging.

### Participants

The volunteers were recruited from the Bilbao area. They were divided into two groups, which were comprised of students and professionals, respectively. The requirement for volunteers to be in the first group was that they had to be in the last or penultimate year of a medical degree in medicine. To be part of the second group, participants were required to have finished their degree in medicine (specialization in neurology or radiology) and be working in a hospital. No distinction was made for gender in either case.

The student group was comprised of 25 individuals: 14 women and 11 men. The average age of this group was 23.64 years old, with a standard deviation of 0.49. The professional group consisted of 25 people, of whom 9 were women and 16 were men. The average age of the group was 49.56 years old, with a standard deviation of 3.25.

All the participants had signed an informed written consent form before the study and were informed of the study purposes and protocols.

### Instruments

As well as the technical instruments described above, a socio-demographic questionnaire and an application survey were used.

In the socio-demographic survey the individual’s age, sex and status (whether they were a professional or a student) were indicated (depending on the inclusion and exclusion criteria). The application survey contained 5 items which were divided into 5 scales (Utility, Design, Usability, Sharing Options and Final Satisfaction). They were completed by using a 5-point Likert-scale where 1 indicated ‘Poor’ and 5 indicated ‘Excellent’. The survey was developed by the authors specifically for this study. The participants were asked to respond to all of the questions, although this was not compulsory. All of the participants responded to all of the questions.

### Procedure

Advertisements for recruitment of volunteers were placed both on social networks and in Faculties of Medicine, hospitals and clinics throughout the Basque Country and Navarra. The advertisements included a telephone number and a contact email address in order to arrange the date for the tests and address or explain any issues the volunteers may have had regarding the survey.

Each volunteer contacted the team, either by email or telephone and full information about the survey was provided and all queries were answered.

In each case, on the agreed date, each volunteer signed an informed written consent form, and then proceeded to use the referred application for at least 15 minutes. There was no specified maximum time length for the test. The volunteers did not have to provide their own terminal or download the application, as the application had been previously installed in a terminal for them. While the application was being used, none of the volunteer’s questions were answered, given that participants’ opinions were being sought on their actual, unaided use of the application, rather than with the help of the application’s designers. At the end of the session the socio-demographic questionnaire and the application survey were completed. In order to control the results of the study, the volunteers were identified by a numeric code that was totally unrelated to any personal data.

### Statistical analysis

The statistical analysis was carried out by using SPSS (IBM Corp. Released 2011. IBM SPSS Statistics for Windows, Version 20.0. Armonk, NY: IBM Corp).

A descriptive analysis was carried out in which both the average and the standard deviation for all the items of the application survey were calculated, separating them into two groups (students and professionals).

Subsequently, a non-parametric inferential analysis was conducted in order to determine mean differences between groups. This analysis was carried out by using the Mann–Whitney *U* test and non-parametric tests, due to the small size of the sample and its limiting normality.

## Implementation

**Figure 1:**
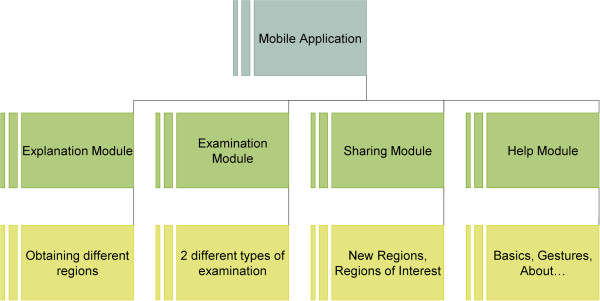
High-level schematic.

In the application there are 4 different modules (Figure 1) that work together to enable users with professional or minimal experience of medical education and knowledge to obtain regions of interest (ROI). All of the modules are necessary and without any one of them, the user’s experience would be greatly diminished.

### Explanation module

**Figure 2:**
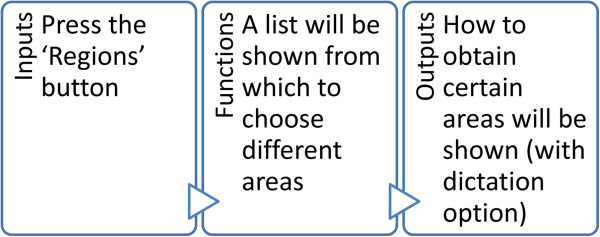
Design of the Explanation Module (high level).

This module contains the most relevant information for the learning of the regions. It constitutes its foundation, as it makes it possible to determine the different regions of interest in any tractography analysis programme of choice (Figure 2).

Different possibilities are offered and the user must choose what is of most interest at each given time. Since there is a large amount of text in some of these (or in the event that there is a problem and the user cannot easily read the text), an option is available for automatic dictation (Text to Speech).

**Figure 3:**
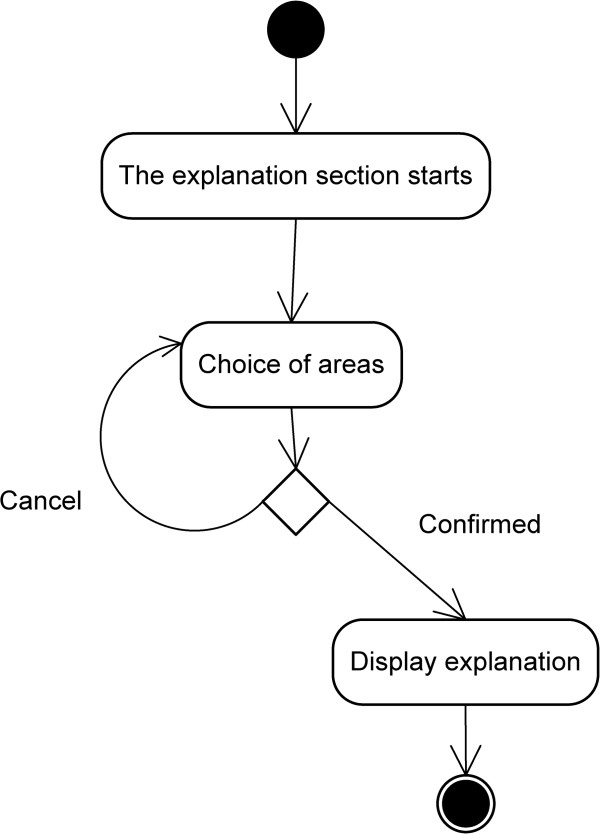
Explanation Activity Diagram.

At the start a scrollable list appears with a large number of options. After selecting the desired option, a small dialogue box appears indicating which region has been chosen and asking for confirmation (whether the user is sure). After confirming (stating ‘Yes, I am sure’), the full explanation of the region in question [20,21] can be seen (at the top of the screen a small menu has been added with a picture of a house, to return to the start [home]) (Figure 3).

### Examination module

**Figure 4:**
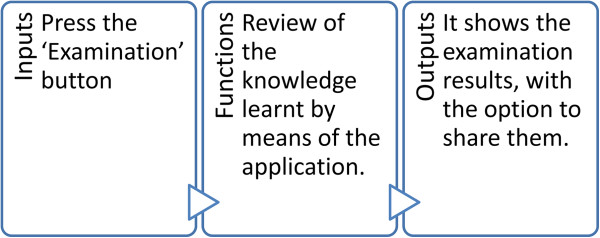
Design of the Examination Module (high level).

An examination module is available for users to test their knowledge (Figure 4). Different screens appear in which the user has to demonstrate that they have a thorough knowledge of the human brain. There are two types of examination, which are interspersed randomly.

➢ *True/False test*→In this test a brain appears on a screen with a region marked by a white circle and a name in the upper part. The user has to draw a ‘G’ (which stands for ‘good’) on the screen if the user thinks that the region matches the name (the letter can be seen as it is being drawn). If the user thinks that the two do not match, a ‘B’ (which stands for ‘bad’) must be drawn on the screen.

**Figure 5:**
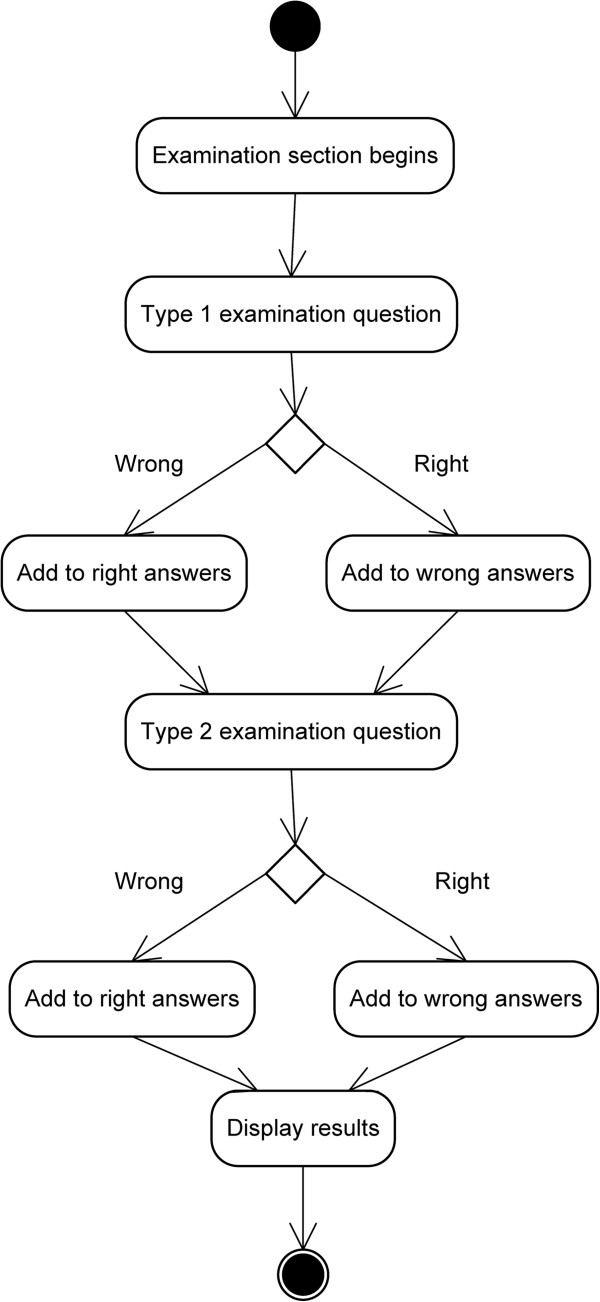
Diagram of examination activities.

➢ *Test to mark the region*→In this type of test a brain appears on the screen without any region marked, but with a name on the upper part of the screen. The user has to indicate by way of a simple touch the region of the brain that is indicated (when a touch is made, a white circle around the region appears).An activity diagram is shown (Figure 5) which describes that there are only two examination questions. Each refers to a different type of examination, to ensure that the diagram can be seen and easily understood.

### Sharing module

**Figure 6:**
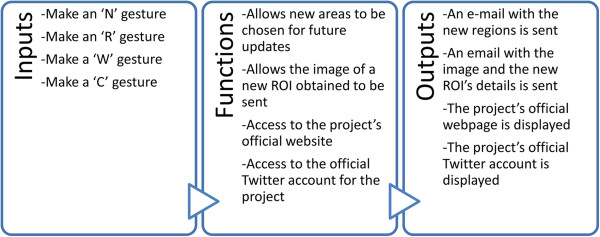
Design of the sharing module (high level).

To access this module the user has to make gestures over the screen of the mobile device, as the main screen only has the two buttons for the explanation and examination modules (Figure 6).

**Figure 7:**
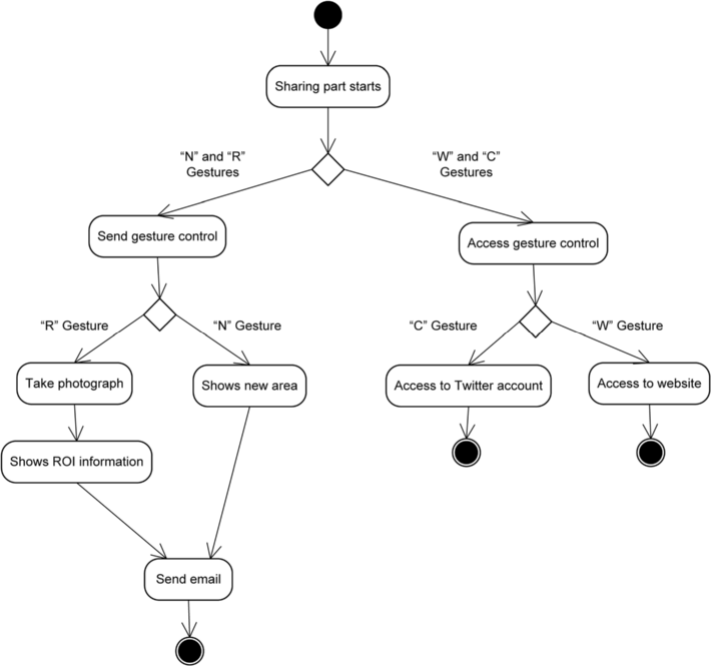
Diagram of sharing activities.

Thanks to this section, suggestions can be made for new regions to be explained, the results obtained can be shared by following the set guidelines (using a photograph), accessing Twitter and the official website…The activity diagram is shown (Figure 7) with the 4 different gestures (each with their corresponding functions) in this module.

➢ * New regions (‘N’ gesture)*→Through this function, the user may indicate which new areas they would like to have in future updates (two options are provided to do this). The new area/s can be indicated by choosing from a list, or by dictating the name of the area/s (by pressing the button with a picture of a microphone).

➢ *Send Region of Interest (‘R’ gesture)*→Drawing this gesture activates the camera to take a photograph of the region of interest (ROI), which is obtained by following the steps outlined in the Explanation Module. After obtaining the image, a scrollable list is used to indicate the specific region and the name of the user (dictating the input by pressing the button with drawing picture of a microphone).

An email will be sent by the email manager chosen by the user. The geo-location option may be activated or de-activated by using the appropriate checkbox.

➢ *Web page (‘W’ gesture)*→Allows the user to easily and quickly access the official webpage. The user will be able to choose the browser to open it.

➢ * Share (‘S’ gesture*)→This action connects to Twitter by means of the official application and with the users previously registered for it (on the official account’s main screen). Once inside, all of the usual functions are available, such as ‘follow’, ‘tweet’, ‘retweet’…

### Help module

**Figure 8:**
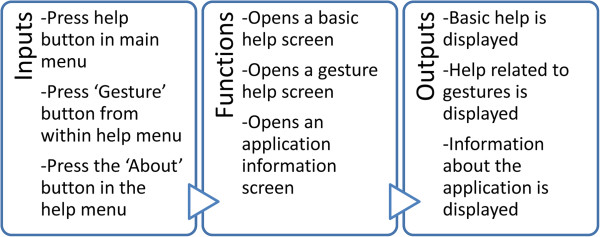
Help module design (high level).

Finally, the help module is found, in which the user can find answers to any questions regarding the project. It is divided into three different sections (Figure 8).

➢ * Basic Help*→This explains the basic use of the application, showing how the main buttons (‘Regions’ and ‘Examination’) work.

➢ * Gesture help*→Leaving the basics to one side, this explains the gestures and how they work in general terms. It is accessed by pressing the ‘Gestures’ button once inside the basic help.

➢ * Developer Information*→Data are provided that are relevant to the programme only, such as who developed it, the year in which it was developed, etc.

**Figure 9:**
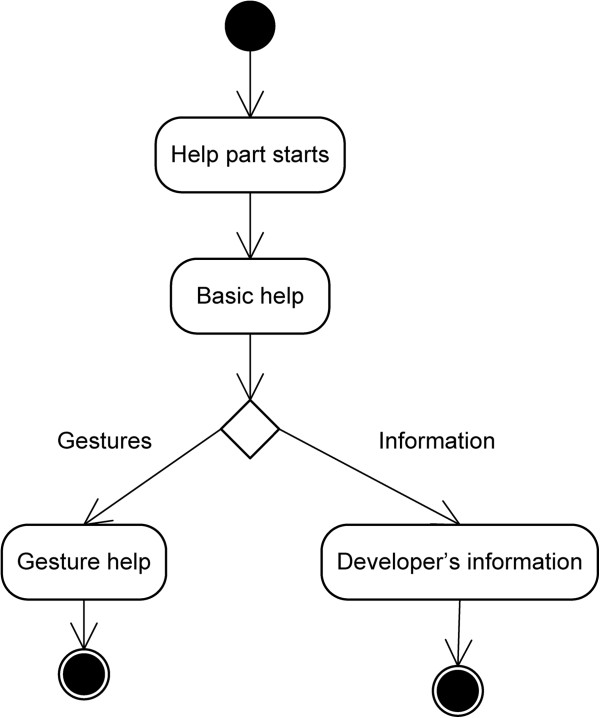
Help Module activities diagram.

The last two options have a small menu with the picture of a house. By pressing this, the user can return to the beginning, thus avoiding having to press the ‘back’ button twice.Once the operation of each of the options in this module has been described, it is important to be aware of the activities diagram for the module, in order to understand how to access the various options (Figure 9).

As can be seen in this diagram, in order to access the gesture help and developer’s information sections, it is necessary to first enter the application’s basic help section.

## Results

**Figure 10:**
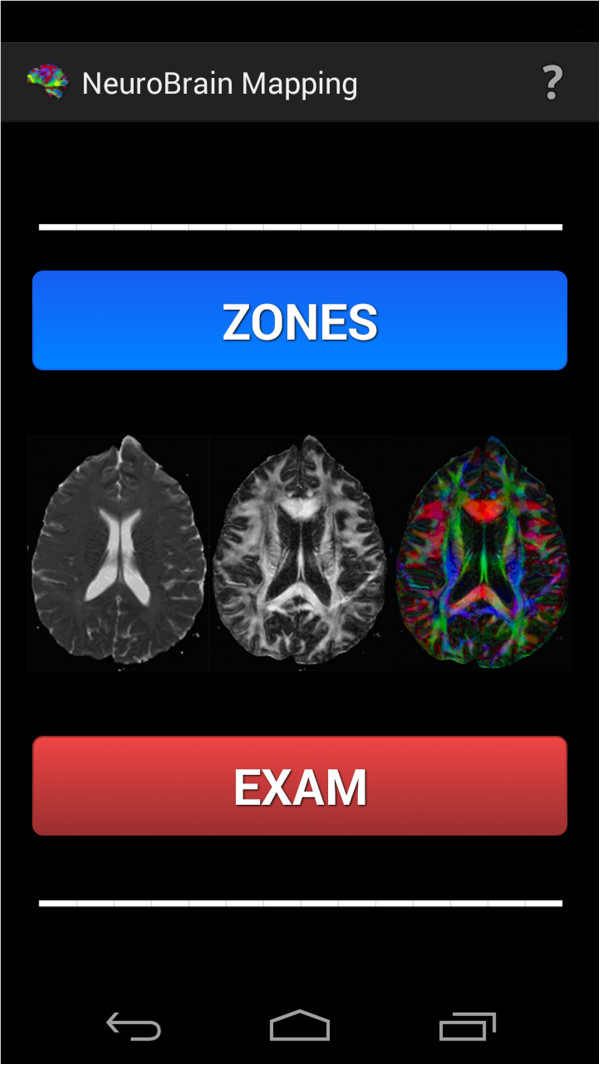
Main Screen.

Once the implementation of the application has been explained, some of its results will be shown below.The first thing to be found is the main screen (Figure 10), with the main buttons and some space in the middle for the gestures described above to be made.

### Examples of use of the tool

**Figure 11:**
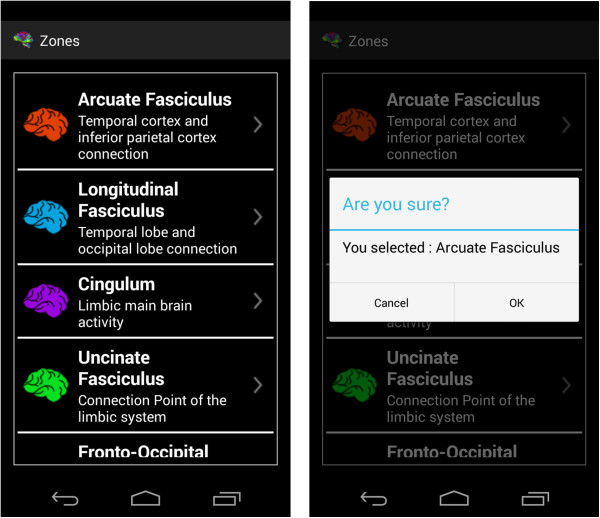
Selection of Regions of Interest.

**Figure 12:**
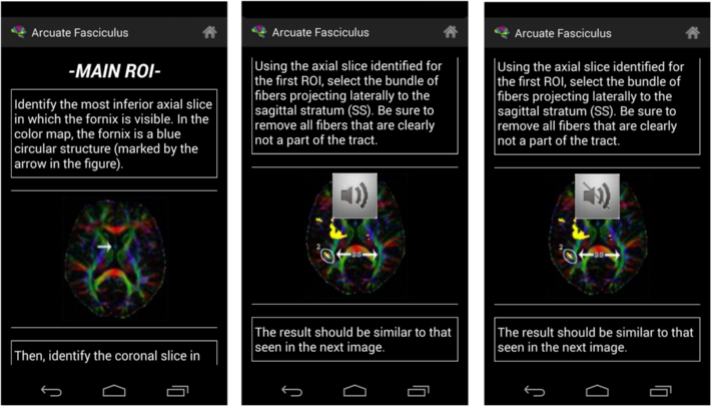
Region of Interest.

To open the explanation of a region of interest, just select it in the available list (Figure 11). Images of multi-colour brains have been added to give a more attractive and minimalist look.After choosing a specific region of interest, it is shown on the screen, together with explanatory images and the appropriate text. If the user wishes the voice help to verbalize the text that appears on the screen, the terminal needs only to be shaken (Figure 12), and it will start to produce speech. The user can silence this voice at any time by merely shaking the device again.

**Figure 13:**
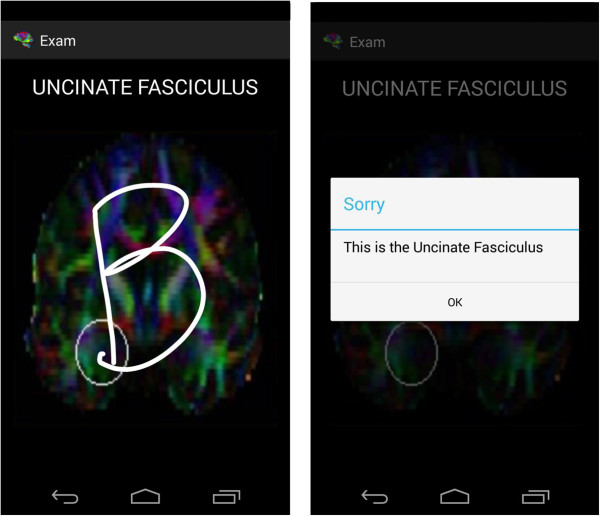
Examination Type 1.

**Figure 14:**
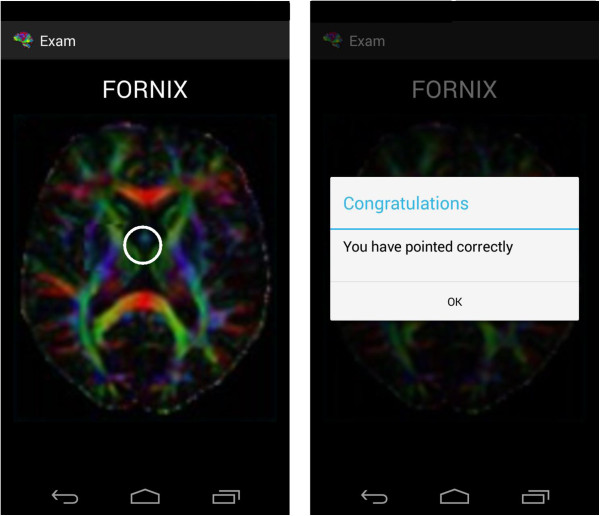
Examination Type 2.

**Figure 15:**
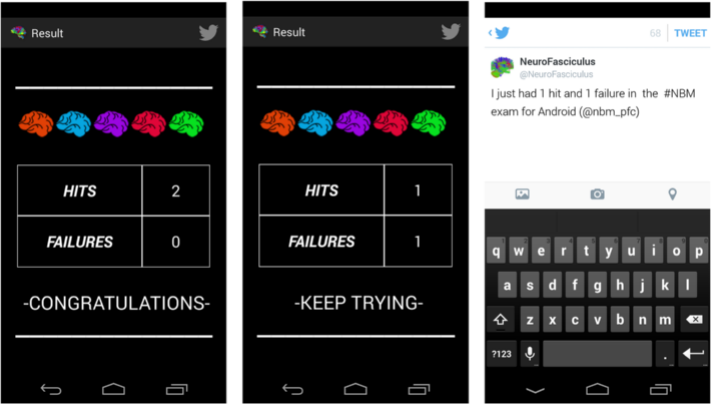
Examination Results.

**Figure 16:**
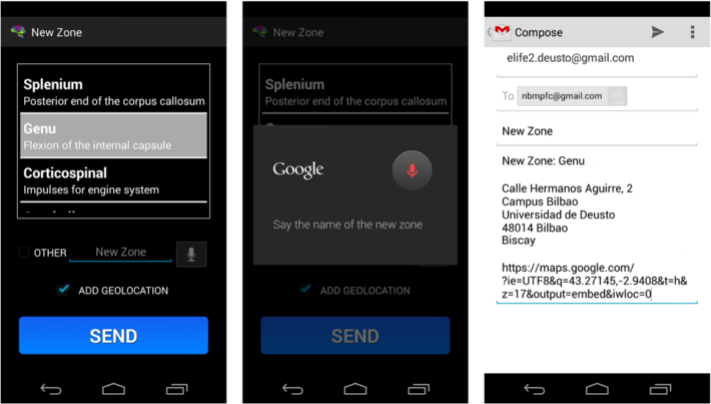
Suggestions of new regions.

**Figure 17:**
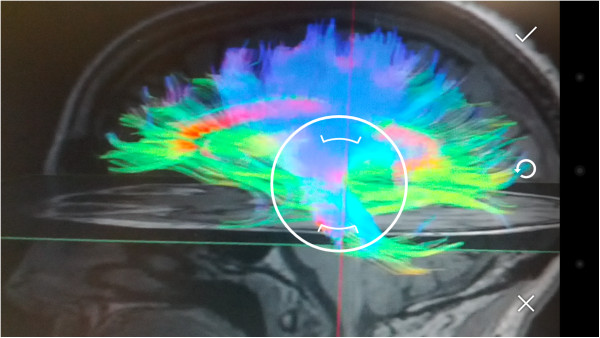
Photograph of a ROI obtained.

**Figure 18:**
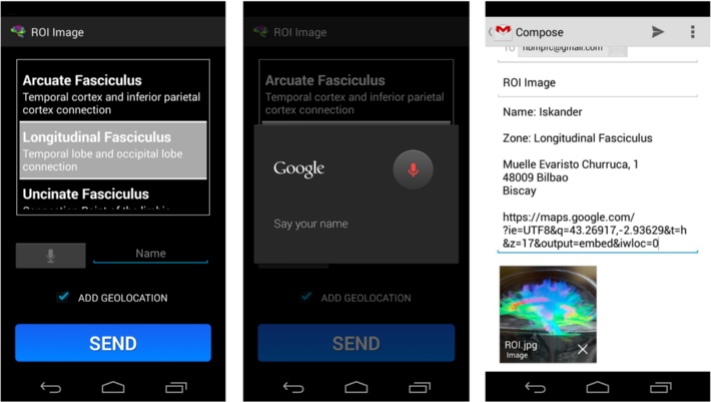
Sharing the Region of Interest (ROI).

**Figure 19:**
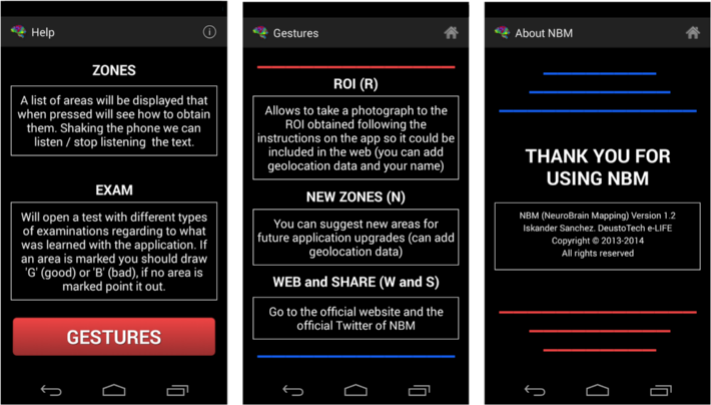
Application Help and Information.

It is important to note that the text converted into speech is designed to start every time the terminal is shaken, which means that the application does not remember where it stopped verbalizing previously. A toast is shown with a speaker symbol or a crossed-out speaker symbol to show what is happening at any given moment.Figure 13 shows an example of the first type of examination and Figure 14 shows an example of the second type of examination. After drawing or marking the desired option, a dialogue appears indicating if the answer is right or wrong (the device also vibrates), to then move to the following examination question.When the user has completed the whole examination, the results can be seen and shared with everyone by means of the social network Twitter (the logo appears in the upper menu). The user does not need to write a message, as it is generated automatically (Figure 15) – it is enough to press the logo if the user wishes to ‘tweet’.Examples of the different application parts of the sharing module can be seen in Figures 16, 17 and 18. It is worth mentioning here that the voice recognition is very good, but if a problem arises that prevents recognition from being done correctly, it is identified as an error and the user can try again.To access the basic explanation, the button with a question mark on it that appears in the menu must be pressed. To access the explanation of the use of gestures, however, the ‘Gestures’ button is to be found in the basic help section, which must be pressed. For information about the application itself, the relevant button found on the basic explanation screen menu must be pressed (Figure 19).

### Application survey

Table 1 shows the results obtained from the descriptive analysis, which details both the average and standard deviation for each item by group.

**Table 1:** Descriptive analysis of the application survey

	**Groups**	**Mean**	**Standard deviation**
Utility	Professionals	3.55	0.605
	Students	4.10	0.718
Design	Professionals	3.90	0.718
	Students	4.10	0.788
Usability	Professionals	3.60	0.821
	Students	4.10	0.912
Sharing Options	Professionals	3.90	0.788
	Students	3.45	0.759
Final Satisfaction	Professionals	4.30	0.470
	Students	4.20	0.410

The results from the non-parametric inferential statistical analysis used to obtain the mean differences between groups, indicate that Utility and Usability show significant statistical differences (p < 0.05), the student group’s average being 3.55 for Utility and 3.60 for Usability, and the professional group’s being 4.10 for both variables. The rest of the variables (design, usability, sharing options and final satisfaction) were not significant (p > 0.05) according to these analyses.

Lastly, it should be noted that the study was widely welcomed by the online community. The official webpage received more than 1000 visits, whilst the Twitter account had more than 280 followers, including a large number of researchers from the fields of neurology and psychology.

## Discussion and conclusion

The development of programmes/applications for PDA terminals has been hampered [22] by the appearance in the market of smartphones, as these offer a greater number of functionalities. The use of mobile technology in learning is very useful, because it allows easy and intuitive access to information [23], as well as providing the option to update information easily.

By way of this application, any student or professional related to medicine can learn how to define specific regions of interest, check their knowledge by using examinations, and share their knowledge with the whole of the scientific community.

Whilst different applications for learning have been designed for Android [24], including specifically for medical training [25,26], currently no other application in the market has the functionalities and facilities provided by the application described in this paper. It allows an analysis and study to be undertaken of different regions of the brain without the need for advanced knowledge of the topic in question. The option to share included in the application, together with the official Twitter account and the website makes it possible to share results and discuss them with other professionals in the sector.

It is important to note that, in order to determine if a person suffers from a specific mental illness, it is a very common procedure to obtain an image of the brain’s white matter, and to calculate various measurements. On many occasions, only the values for specific areas of the brain are of interest, and it is in these situations that this application is used, as it allows the desired areas of the brain to be identified. Once the desired area has been correctly defined, the calculation of the relevant measurements can be made. These will show whether or not the person suffers from a particular mental illness. The application therefore streamlines the analysis of the brain’s white matter to a large extent.

The analysis of the application survey shows marked differences in the results between the groups, but this is understandable. Still, both groups stated that they were very satisfied in the final assessment of the application and wrote very positive and encouraging comments. The students showed themselves to be satisfied with all the sections of the application, except with the option for sharing, which had a lower score. It is usual for students not to consider this option to be very useful, as they are more concerned with the knowledge they gain themselves than with sharing it with the neuro-scientific community. As to professionals, they declared to be less satisfied in terms of utility and usability, as they considered that they already knew part of the information in the application, and they are less used to working with new technologies. However, they ranked the design and sharing options highly, as these are very useful in the professional scientific world.

With respect to future lines of research, some additional facilities are being considered that could somewhat improve the application and increase its prospects. Regarding the future of the application, it must be considered that the mobile operating system market is very fragmented (although the undisputed leaders are Android and iOS), and the application is currently only available for one of them [27,28]. For this reason, it would be appropriate to develop an application for Apple products as well. As this system has a ‘personal assistant’ called Siri, which has an ever-increasing strength in the market, it would be beneficial for the application to be completely integrated into it, as this would give users a completely new experience.

In the current version of the application no specific customisation was made for either group (students or professionals). But based on the results obtained from the application survey, one version could be developed for each group, in order to improve the utility capabilities for students, and the sharing options for professionals.

It also has to be taken into account that, given that the application allows suggesting new regions (by using a predetermined list or by dictating a specific region), these regions will be included in future updates. In this way two aims are achieved: firstly, the users will see that their contributions and recommendations are taken into account, and secondly, the application will be increasingly more complete and will have a wider range of areas available.

## Availability and requirements

Project name: Mobile NBM.

Project download page: http://goo.gl/Uf936b.

Operating system(s): Android OS.

Programming language: Java (using the Android SDK).

Any restrictions to use by non-academics: Author’ permission.

## Abbreviations

PDA: Personal digital assistant; ROI: Region of interest; MRI: Magnetic resonance imaging; TTS: Text to speech; SR: Speech recognition; GPS: Global positioning system.

## Competing interests

The authors declare they have no competing interests.

## Authors’ contributions

ISR developed and tested the application and wrote the manuscript. BGZ tested the application and reviewed the manuscript. Both authors have read and approved the final manuscript.

## Pre-publication history

The pre-publication history for this paper can be accessed here:

http://www.biomedcentral.com/1472-6920/14/148/prepub
